# Off-label intravitreal eculizumab for geographic atrophy: report of the first two cases

**DOI:** 10.1186/s40942-026-00842-1

**Published:** 2026-04-06

**Authors:** Rodrigo Jorge, Marcelo G. B. Rego, Arthur S. Zupelli, Rodrigo T. Calado

**Affiliations:** 1 Ophthalmology Division, Ribeirão Preto Medical School, University of São Paulo, Ribeirão Preto, SP Brazil; 2 Department of Medical Imaging, Hematology and Clinical Oncology, Ribeirão Preto Medical School, University of São Paulo, Ribeirão Preto, SP Brazil

**Keywords:** Eculizumab, Age-related macular degeneration, Geographic atrophy

## Abstract

**Purpose:**

To report the feasibility and short-term safety of off-label intravitreal eculizumab as a complement-inhibition strategy for geographic atrophy (GA) secondary to age-related macular degeneration (AMD), based on two clinical cases.

**Case reports:**

Two pseudophakic patients with progressive bilateral visual decline presented with GA and drusen in both eyes. Because this represents a novel, off-label therapeutic approach, intravitreal eculizumab was administered first in the worse-seeing eye in both cases, which also exhibited more advanced atrophic involvement. The treatment was selected as a potentially more accessible alternative to pegcetacoplan or avacincaptad pegol, which remain unavailable in Brazil. At fourmonth follow-up, no ocular toxicity was observed. There was no reduction in visual acuity in the treated eyes; in Case 1, BCVA improved from 20/400 to 20/200, while Case 2 maintained baseline acuity. Full-field electroretinography performed at baseline and at month 1 demonstrated preserved responses without reduction in B-wave amplitude, and multifocal electroretinography showed stable response densities with no amplitude loss.After functional assessment, structural multimodal imaging also showed no evidence of toxicity. Optical coherence tomography (OCT) revealed stable GA-related outer retinal atrophy without inner retinal changes, intra- or subretinal fluid, or any signs of acute retinal injury. Fundus autofluorescence and OCT angiography remained unchanged, with no findings of ocular inflammation, vascular compromise, or early acceleration of GA progression.

**Conclusions:**

Intravitreal eculizumab was feasible and demonstrated short-term safety in these two cases, with preserved visual function and no electrophysiological or structural evidence of toxicity. While biologically plausible as a C5-inhibition strategy, its therapeutic benefit remains unproven. Larger, controlled studies with extended follow-up are required to clarify long-term safety and efficacy in geographic atrophy.

## Introduction

Age-related macular degeneration (AMD) is the leading cause of vision impairment among individuals aged 65 years and older. While patients with neovascular (wet) AMD have benefited over the past two decades from the effectiveness of anti-VEGF therapy, those with late-stage dry AMD—particularly geographic atrophy (GA)—have only recently gained access to pharmacologic interventions. GA is characterized by progressive degeneration of the outer retina, retinal pigment epithelium, and choriocapillaris, ultimately resulting in irreversible and debilitating visual loss.

In February 2023, pegcetacoplan became the first US Food and Drug Administration (FDA)-approved therapy for GA, acting through inhibition of C3 and C3b within the complement cascade [1, 2]. Later in 2023, avacincaptad pegol—an inhibitor of complement factor C5—also received FDA approval [3, 4]. Although these agents represented a major therapeutic advance, their international market prices remain extremely high, with estimated per-injection costs of approximately USD 2,000. Such pricing renders large-scale or long-term treatment with these drugs unfeasible within the Brazilian public health system and limits accessibility even in private care, where continuous monthly or bimonthly dosing imposes substantial cumulative financial burden.

Eculizumab (Soliris), a monoclonal antibody targeting complement component C5, was the first drug to inhibit the terminal complement pathway [5–8]. Although approved for systemic use in conditions such as paroxysmal nocturnal hemoglobinuria, atypical hemolytic uremic syndrome, and myasthenia gravis, its commercial formulation allows for dose fractionation. Notably, when subdivided, the cost per intravitreal dose may be reduced to approximately USD 40—rendering it dramatically more affordable than currently approved GA treatments.

Given its established mechanism of complement inhibition, biological plausibility for GA, and markedly lower cost when fractionated [5–8], our group sought to evaluate the short-term ocular safety of intravitreal eculizumab in patients with geographic atrophy secondary to age-related macular degeneration in a setting where no approved therapies are currently available.

## Case report 1

**Fig. 1:**
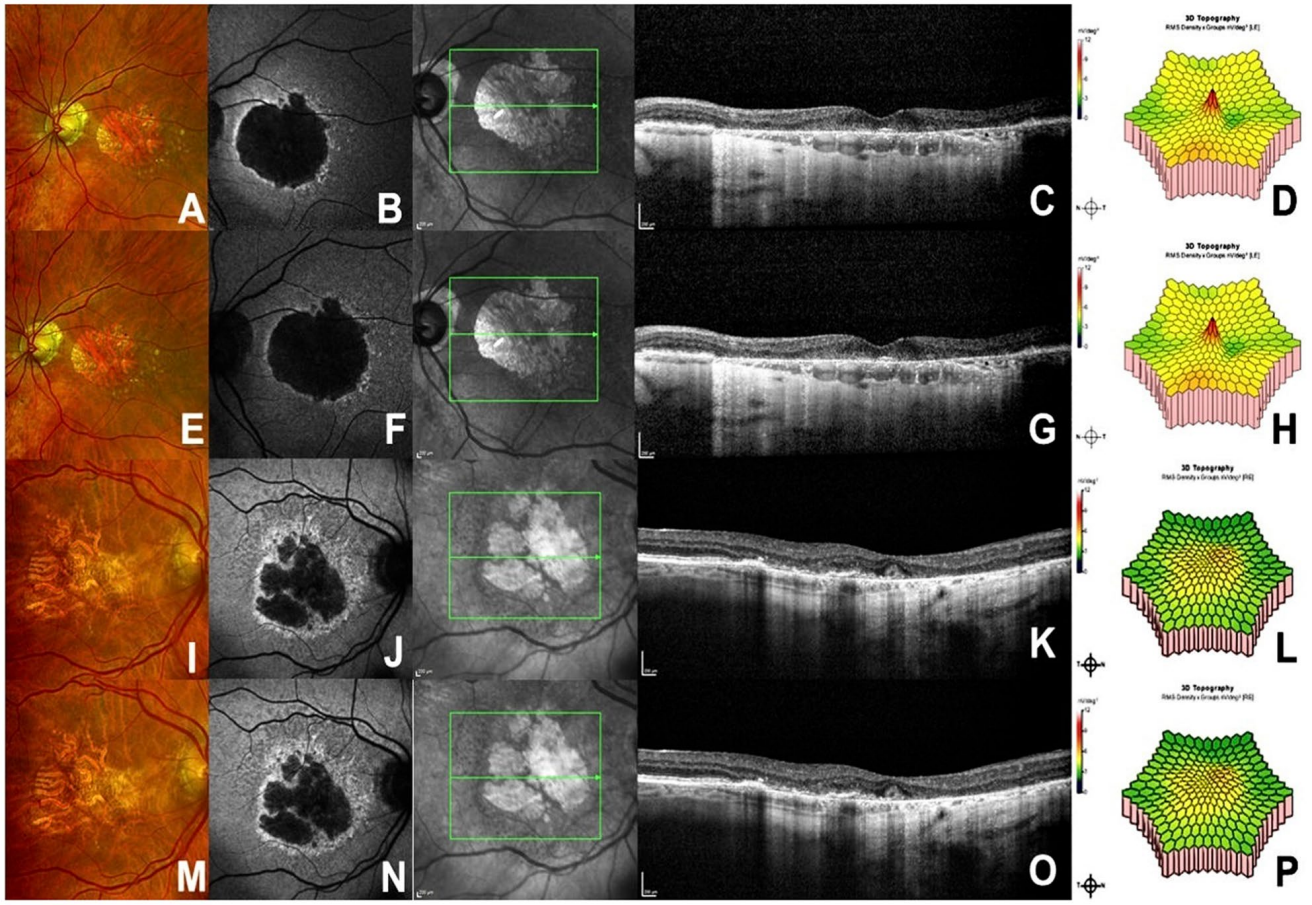
Multimodal imaging findings before and after intravitreal Eculizumab (1.0 mg/mL) in the first two patients. **A** and **H** (Patient 1). (**A**, **E**) Ultra-widefield color fundus images showing central geographic atrophy with drusenoid deposits before and one month after intravitreal eculizumab. (**B**, **F**) Fundus autofluorescence demonstrating widespread macular hypoautofluorescence corresponding to areas of retinal pigment epithelium (RPE) loss. (**C**, **G**) Structural OCT displaying subretinal drusenoid material, outer retinal thinning with a waterfall-like configuration, and diffuse RPE atrophy consistent with areas of complete RPE and outer retinal atrophy (cRORA). (**D**, **H**) Multifocal electroretinography at baseline and one month post-treatment showing stable retinal sensitivity without functional decline. **I** and **P** (Patient 2). (**I**, **M**) Ultra-widefield color fundus photographs revealing extensive macular geographic atrophy before and one month after intravitreal eculizumab. (**J**, **N**) Fundus autofluorescence showing diffuse macular hypoautofluorescence with no detectable interval progression on short-term follow-up. (**K**, **O**) OCT B-scans demonstrating subretinal drusenoid deposits, outer retinal disruption with a cascading pattern, and diffuse RPE atrophy involving regions of incomplete (iRORA) and complete (cRORA) RPE and outer retinal atrophy. (**L**, **P**) Multifocal electroretinography at baseline and one month post-treatment demonstrating preserved retinal function without amplitude reduction

A 77-year-old pseudophakic man with a five-year history of progressive bilateral visual decline presented with BCVA 20/50 OD and 20/400 OS. Anterior segment examination was unremarkable. Color fundus pictures revealed bilateral geographic atrophy (GA) with soft confluent drusen, more advanced in OS. Structural OCT revealed prominent drusen and subretinal drusenoid deposits and well-demarcated areas of complete outer retinal atrophy (cRORA). In OS, the atrophy fully involved the foveal center, with collapse of the outer retinal layers and loss of the ellipsoid and interdigitation zones. In OD, cRORA was also present but only partially encroached upon the foveal center, leaving a residual island of preserved outer retinal structure. OCT angiography excluded macular neovascularization. Fundus autofluorescence demonstrated macular hypoautofluorescence consistent with GA. (Figure 1A and C). After extensive discussion with the patient, and because approved complement inhibitors such as pegcetacoplan and avacincaptad pegol are not available in Brazil, and GA progression was more advanced in OS, off-label intravitreal eculizumab (1 mg/0.1 mL) was administered in OS after informed consent.

At one-month follow-up, multifocal electroretinography showed preserved central and perifoveal response densities, without amplitude reduction. Full-field ERG similarly demonstrated normal scotopic and photopic responses with preserved B-wave amplitude. BCVA improved to 20/200. No ocular inflammation, IOP elevation, uveitis, vasculitis, or other adverse effects were observed. Multimodal imaging revealed no structural changes suggestive of toxicity (Fig. 1D and H), with stable inner retinal layers and unchanged atrophic patterns (Fig. 1E and H). At the four-month follow-up, following four monthly intravitreal injections, clinical and multimodal imaging findings remained unchanged, with no evidence of disease progression or treatment-related toxicity.

### Case report 2

An 89-year-old pseudophakic woman with a 15-year history of progressive visual decline presented with BCVA 20/400 OU. Anterior segment examination was normal. Multimodal imaging revealed extensive bilateral GA with soft drusen and greater foveal involvement in the right eye. Structural OCT revealed prominent subretinal drusenoid deposits accompanied by well-defined areas of incomplete (iRORA) and complete (cRORA) outer retinal atrophy throughout the macula. These atrophic changes were more extensive in the right eye, where they showed deeper outer retinal collapse, greater disruption of the ellipsoid and interdigitation zones, and broader confluent involvement compared with the fellow eye. Fundus autofluorescence confirmed diffuse macular hypoautofluorescence, and OCT angiography showed no macular neovascularization. (Figure 1I and K). After extensive discussion with the patient, and given the progression of GA, the role of complement dysregulation, and the unavailability of pegcetacoplan or avacincaptad pegol in Brazil, off-label intravitreal eculizumab (1 mg/0.1 mL) was administered in the worse-seeing right eye after informed consent.

At one month, multifocal electroretinography revealed stable response densities without amplitude loss (Fig. 1L and P). Full-field ERG demonstrated preserved scotopic and photopic responses with normal B-wave amplitude. BCVA remained stable. There were no signs of ocular inflammation, persistent conjunctival hyperemia, IOP elevation, anterior chamber reaction, uveitis, or retinal vasculitis. Follow-up multimodal imaging showed no structural evidence of toxicity (Fig. 1M and P). Also, for this patient, following four monthly intravitreal injections, clinical and multimodal findings remain unchanged at four-month follow-up visit.

## Discussion

Recent advances in complement inhibition have demonstrated that slowing the progression of geographic atrophy (GA) is feasible. Pegcetacoplan (Syfovre^®^), a C3 inhibitor, showed statistically significant reductions in GA enlargement in the phase 3 OAKS and DERBY trials. At 12 months, monthly pegcetacoplan reduced lesion growth by 22% in OAKS and 18% in DERBY, while every-other-month dosing achieved 16% and 17% reductions, respectively [2]. At 24 months, these effects were further amplified, with monthly dosing slowing progression by 36% in OAKS and 29% in DERBY [2]. Considering that untreated GA typically enlarges at approximately 1.5–2.0 mm²/year, these reductions correspond to an absolute decrease of roughly 0.3–0.6 mm²/year in lesion growth. Similar results had been demonstrated in the phase 2 FILLY trial [1].

Similarly, avacincaptad pegol (Izervay^®^), a C5 inhibitor, demonstrated meaningful benefit in the GATHER trials. In GATHER1, monthly 2-mg avacincaptad pegol reduced GA growth by 27% at 12 months [3], whereas in GATHER2, monthly intravitreal administration of 2 mg (every 28 days) slowed progression by 14% at one year [4], translating to an approximate reduction of 0.2–0.4 mm²/year in lesion enlargement. Together, these pivotal trials validate complement inhibition—either at C3 or C5—as a clinically effective strategy capable of reducing structural progression of GA [1–4].

Eculizumab is a humanized IgG2/4κ monoclonal antibody that inhibits complement component C5 by preventing its cleavage into C5a and C5b, thereby blocking formation of the membrane attack complex (MAC, C5b-9) and terminal cytolytic activity [5–8]. Originally developed for paroxysmal nocturnal hemoglobinuria (PNH), where its potent complement inhibition has proven highly effective [5, 6], eculizumab offers a strong biological rationale for investigation in GA, a condition in which terminal complement activation plays a central pathogenic role. This mechanistic alignment is strengthened by the fact that avacincaptad pegol—the most recently approved therapy for GA—exerts its therapeutic effect through the same C5 inhibition pathway [3, 4]. Thus, the well-established systemic efficacy of Soliris^®^ in suppressing C5 activation supports the rationale for exploring localized intravitreal administration of fractionated eculizumab as a potential complement-modulating approach in GA [5–8].

However, the systemic use of eculizumab for GA failed to demonstrate efficacy in the randomized COMPLETE study, despite producing full systemic complement blockade. A central limitation of systemic delivery is pharmacokinetic: eculizumab, as a large IgG2/4κ monoclonal antibody (~ 148 kDa), penetrates the blood–retinal barrier extremely poorly, resulting in vitreous and retinal concentrations far below therapeutic thresholds. High-impact pharmacokinetic studies demonstrate that systemically administered IgG molecules typically achieve well under 1% of serum concentrations in the vitreous and retina—often below detectable levels—making meaningful intraocular complement suppression unlikely [9]. The COMPLETE trial also included a very small sample—only 10 patients receiving high-dose IV eculizumab, 10 receiving low-dose, and 10 controls. Even if a minute fraction of the drug were to reach the vitreous cavity, the expected effect size on GA progression would be extremely small due to subtherapeutic intraocular exposure. Such a marginal treatment effect would be statistically undetectable in a trial with only 10 participants per arm. Therefore, the negative results of COMPLETE more plausibly reflect the pharmacokinetic limitations of systemic administration rather than an absence of therapeutic potential for C5 inhibition in GA. These considerations further support bypassing systemic delivery and directly investigating intravitreal fractionated eculizumab as a strategy to achieve pharmacologically relevant local C5 inhibition [10]. In this report, we used an intravitreal dose of Eculizumabe 1 mg/0.1 mL, approximately half of the FDA-approved per-injection dose of avacincaptad pegol (2 mg) used in the GATHER trials [3, 4]. Because Izervay^®^ may be administered monthly or every two months, a regimen of 1 mg of intravitreal eculizumab administered monthly would theoretically approximate the cumulative 2-mg bimonthly exposure used in the approved dosing schedule. This proportional-dose framework provides pharmacologic coherence and supports the exploration of fractionated intravitreal eculizumab as a feasible C5-blocking strategy for GA.

Despite strong mechanistic plausibility, eculizumab is not approved for ophthalmic use, and both pegcetacoplan and avacincaptad pegol remain inaccessible in Brazil as of 2025 due to their high international costs and lack of commercial availability. Even in countries where these agents are marketed, the substantial cumulative cost associated with lifelong treatment creates barriers to access and widens disparities in GA management, particularly in low-resource settings.

From a public-health perspective, the development of accessible, lower-cost complement-modulating therapies is essential. A single commercial 30-mL vial of Soliris^®^ can be fractionated into approximately 250–300 intravitreal doses of 1 mg/0.1 mL, reducing the cost per dose to roughly USD 40. This dramatic reduction carries profound social implications: one vial could treat an entire regional population of GA patients in a public-health setting.

In our two cases, intravitreal eculizumab was well tolerated, with no signs of ocular inflammation, intraocular pressure elevation, uveitis, vasculitis, structural abnormalities, during four-months follow up. No electrophysiological changes were observed during one-month follow-up. These findings align with the short-term ocular safety profile reported for other complement inhibitors [1–4].

This study has clear limitations, including the small number of patients and short follow-up duration, which restrict conclusions about long-term safety or efficacy. Nonetheless, given the absence of accessible GA treatments in Brazil, the prohibitive cost of existing complement therapies, and the enormous potential socioeconomic benefit of a low-cost fractionated alternative, this initial clinical experience is highly relevant. As the first report describing intravitreal fractionated eculizumab for GA, these findings open an important path for future research. Larger, controlled studies are needed to determine whether this approach can safely slow GA progression in populations otherwise unable to access high-cost biologic therapies.

## Data Availability

All data are included within the article.
