# Correction to: Off-label intravitreal eculizumab for geographic atrophy: report of the first two cases

**DOI:** 10.1186/s40942-026-00894-3

**Published:** 2026-06-29

**Authors:** Rodrigo Jorge, Marcelo G. B. Rego, Arthur S. Zupelli, Rodrigo T. Calado

**Affiliations:** 1 Ophthalmology Division, Ribeirão Preto Medical School, University of São Paulo, Ribeirão Preto, SP Brazil; 2 Department of Medical Imaging, Hematology and Clinical Oncology, Ribeirão Preto Medical School, University of São Paulo, Ribeirão Preto, SP Brazil

**Correction to: International Journal of Retina and Vitreous 2026;54(12)**

10.1186/s40942-026-00842-1

In this article, the ethical approval statement was missing and should be included as follows.

The authors confirm that this study was approved by the Brazilian National Research Ethics Committee (Comissão Nacional de Ética em Pesquisa – CONEP), under CAAE number 87450325.1.0000.5440.

The original article has been corrected.

